# Care pathways in the transition of patients between district psychiatric hospital centres (DPCs) and community mental health services

**DOI:** 10.1002/hsr2.37

**Published:** 2018-04-10

**Authors:** Eva W. Sather, Marit F. Svindseth, Paul Crawford, Valentina C. Iversen

**Affiliations:** ^1^ Faculty of Medicine and Health Sciences Norwegian University of Science and Technology Trondheim Norway; ^2^ Faculty of Medicine and Health Science Norwegian University of Science and Technology Aalesund Norway; ^3^ Faculty of Medicine and Health Sciences University of Nottingham UK; ^4^ Faculty of Medicine and Health Sciences, Department of Mental Health Norwegian University of Science and Technology, St Olav's University Hospital HF, Tiller District Psychiatric Centre Trondheim Norway

**Keywords:** care pathways, communication, community mental health care, district psychiatric centre, information, integrated care, patient transition, psychiatric services

## Abstract

**Rationale, aims, and objectives:**

Patients with mental health problems experience numerous transitions into and out of hospital. Primary care providers have mixed success in identifying and managing patients' needs. This study explores health personnel's experience of care pathways in patient transition between inpatient and community mental health services.

**Methods:**

A descriptive qualitative design was chosen. Four focus group interviews with 12 informants from 7 different communities were conducted. Interviews were analyzed thematically.

**Results:**

Two main themes were identified: integrated care and patient activation. The participants shared their experiences on topics that can affect smooth care pathways in mental health. Six promoting factors were identified for successful patient transition: opportunities for information sharing, implementation of systematic plans, use of e‐messages, around‐the‐clock care, designating one responsible health person in each system for each patient, and the involvement of patients and their families. The following barriers were all found to impede the patients' transition between levels of care: the lack of a single responsible person at each health care level, insufficient meetings, the absence of systematic plans, difficulties in identifying the right staff at different levels, delays in information sharing, and the complexity of welfare systems negatively affecting patient dignity.

**Conclusions:**

Systems and procedures should be developed to ensure clear responsibilities and transparency at each stage of the pathways of care. A single person should take charge of ensuring sufficient connection and communication between inpatient and community mental health services. Finally, both patient and staff in community services should be linked through a direct telephone number with around‐the‐clock availability.

## 1 INTRODUCTION

Patients with mental health problems experience numerous transitions into and out of hospital.^1^ Evidence shows that patients with mental health concerns often share their problems with their primary‐care provider^2^, ^3^ but that primary care providers have mixed success in identifying and managing these concerns on their own.^4^, ^5^ Because patients have a variety of preferences for care and face barriers associated with mental health treatment, this situation suggests the need for easy access to a range of treatments and providers.^6^, ^7^

There is a growing interest in extending care pathways in primary care and mental health to improve the quality of care through enhanced care coordination. Care pathways are understood as interventions for the care management of mental health patients in need of complex health services during a well‐defined period of time.^8^ Although there seems to be a consensus on the importance of early intervention in the treatment of mentally ill patients,^3^ evidence is sparse about the relationship between care pathways and care coordination. A recent study^9^ found that care pathways are effective interventions for enhancing teamwork, elevating the organizational level of care processes, and reducing the risk of burnout for health care teams in such settings. From care pathways, high‐performance teams can be built.^9^ Chew‐Graham et al^10^ pointed out that, depending on its quality, communication could function as both a promoting factor and a barrier to success. Starfield^11^ identified the following key elements in the integrative functions of primary care: First Contact Care (use of services for each new problem), Continuous Care (regular source of care over time), Comprehensive Care (availability of a range of services), and Coordinated Care (linking of health care events). These 4 elements are implicitly incorporated in the health care system to improve outcomes.^12^ Vickers et al^13^ noted that expanding integrated mental health care in the primary care setting/services resulted in increased staff and provider satisfaction.

A study^14^ evaluating the effectiveness and satisfaction outcomes of a mental health screening and referral clinical pathway for community nursing care showed that the use of a structured pathway by generalist community nurses may result in better recognition and management of problems compared with nurses' reliance on judgment alone. When studying how a care pathway model works in community mental health in the UK, Khandaker et al^15^ found that it led to more focused interventions being offered. However, Steinacher et al^16^ investigated the changes due to the implementation of care pathways in the treatment of patients with schizophrenia and found that the patients reported less treatment satisfaction after the implementation of pathways of care. Steinacher et al offered no explanation, and the evidence base for such pathways remains contested or in development. Katschnig,^17^ for example, emphasized the importance of monitoring different levels of health care to find the best models or pathways of care. Waters et al^18^ suggested that documentation does not reflect patients' views on treatment. However, several studies have revealed that care pathways improve the components of care coordination.^19^, ^20^

A main element in the Coordination Reform in Norway,^5^, ^21^ relevant for the current study, is the commitment to ensuring that patients receive the most effective health care services possible, through cohesive and integrated care pathways, and recommends a 24‐hour follow‐up in the community after discharge from the hospital.

The apparent goal of care pathways is to achieve optimal efficiency and improve the quality of care as prioritized in health strategies in Norway. Thus, the current study endeavors to contribute to this area of research by exploring community health personnel's experience and providing an understanding of care pathways in the patient transition between district psychiatric centres (inpatient) and community mental health services.

## 2 METHODS

To reveal important factors in care pathways for mental‐health patients, we used a http://topics.sciencedirect.com/topics/page/Qualitative_research design with a descriptive approach.^22^

The interviews were conducted in 4 focus groups. Prior to the focus group sessions, we discussed in great depth which questions to ask. We studied the comprehensive summaries of phenomena and events described in the focus group sessions in an effort to detect major categories, themes, and patterns, using thematic analysis.^23^, ^24^, ^25^

### 2.1 Process of selection of participants

The team leaders in the community health care settings identified experienced mental health personnel. All the leaders were positive about the study and acknowledged the need for focusing on pathways of care, especially obstacles that could prevent smooth transitions. They assisted the researchers in identifying participants who would offer comprehensive and unbiased information. All our participants were involved in practical coordination in pathways of care. The inclusion criteria were > 5 years of experience in mental health care and working at least 30 hours a week.

### 2.2 Participants and demographics

Twelve health employees from 7 community health care settings (1 urban and 6 rural) were interviewed in 4 focus groups. All participants were female with more than 10 years of experience in mental health. The vast majority of health personnel in mental health in Norway are women. The study included 9 nurses, 2 carers, and 1 social worker, all specialized in mental health care.

### 2.3 Ethics

The study was approved by the Norwegian Centre for Research Data (NSD, project no. 51960) with no additional approval required for ethical clearance. All phases of the study were conducted according to the Helsinki Declaration^26^ and ethical principles in research. Data were transcribed and anonymized accordingly. Written consent was obtained from all participants.

### 2.4 Focus group interviews

We used a semi‐structured interview guide in the focus group interviews, which was developed in discussion with university and health care representatives. The participants were asked to describe their views on experiences with care pathway transitions between DPCs and community mental‐health services. The interviewer guided the focus group discussion according to the following topics: planning; cooperation between patient and staff; patient participation; ethical issues; communication including information‐giving and documentation in all settings; clinical care and treatment; medication; interdisciplinary cooperation; and organization of information among health personnel. An assistant moderator contributed by regularly summarizing and following up on key information revealed in the group discussions.^27^, ^28^ At the end, we asked general open‐ended questions to gather information that had previously not been expressed.

All interviews were audiotaped and transcribed verbatim. The duration of each focus group interview was between 90 and 120 minutes.

### 2.5 Data analysis

Interviews were transcribed and analyzed through thematic text analysis in 6 phases: familiarizing ourselves with the data, coding, searching for themes, reviewing themes, defining and naming themes, and writing up.^29^ A codebook was developed on the basis of variables identified by our research team at the beginning of the study as theoretically relevant to the research questions and the literature. Graneheim and Lundman's^30^ proposed measures of trustworthiness (credibility, dependability, and transferability) were applied throughout the steps of the research procedure. The analysis of group‐level data involved scrutinizing the themes, interactions, and sequences within and between groups. We performed an iterative analysis in a systematic, repetitive, and recursive process.

## 3 RESULTS

Two areas of concern about care pathways between DPCs and community mental health services emerged from the analysis: (1) the need for integrated care and (2) the need for patient activation or empowerment. These 2 areas are discussed below.

No particular differences between participants from rural and urban health care were found.

### 3.1 Integrated care

Integrated care occurs when health care professionals consider all health conditions at the same time, instead of adopting a fragmented, disease‐specific focus. Thus, integrated treatment is more likely to be customized to individual patients, because this approach allows health care professionals to treat individual patients as a whole rather than on the basis of their separate conditions. Different dimensions play complementary roles: clinical integration, professional and organizational integration, and system integration.^12^

The community mental health teams emphasized the importance of capitalizing on opportunities for cooperation, through the establishment of routine meetings between staff in DPCs and community services to exchange information and to provide quality health care, as stated in the Norwegian government's goals for mental health care.^5^

“We always have the patient's consent to share information. I think that it is necessary to secure cooperation with the most important authorities, particularly in the transitional period from one organizational system to another.”

Some of the participants emphasized a positive change associated with the establishment of routine meetings at inpatient facilities. Before admission to a hospital‐based service, patients were offered to be part of the planned inpatient‐stay program. Participants pointed out the benefit of holding this new routine meeting.

“It seemed to be a very positive experience for the patient; she became more motivated to accept mental health hospitalization. Her contact specialist nurse considered the meeting as goal‐oriented and emphasized that the patient had the opportunity to talk about her challenges.”

One of the participants recommended implementing knowledge‐based protocols for meeting patients prior to their discharge from inpatient settings. She described the current situation as follows:

“Sometimes, we do not have time for a meeting prior to discharge, and we get the information by phone. There are no routines for phone calls or meetings. Different nurses choose different ways of communicating.”

The lack of standardized protocols seemed to preoccupy our participants, and they suggested several ways to facilitate the seamless exchange of important information between systems. The importance of providing and receiving correct information at the right level and time is described in a previous study,^31^ which reviewed evidence on the quality of information transfer between primary care physicians and specialist mental health providers for referral and after inpatient discharge. Previous research has also revealed variability in the quality of protocols in mental health care, with differences existing between regions and among providers and, in some cases, a lack of correspondence between the provided care and the standards of evidence‐based mental health care.^32^

Participants emphasized the need for new evidence‐based protocols for the patient discharge process. One staff member succinctly expressed this shared sentiment when she made the following remark:

*“I think DPCs need routines for the discharge process.”*

Participants from community mental health services were pleased with the hospital‐based meetings about the transfer of patients to community mental health services, but they noted that the information provided by the hospitals was sometimes incomplete. They felt that the delivery of complete patient information by the DPC should be a matter of standard practice when patients return home and the responsibility for their well‐being shifts to the community mental health services. The historical documentation from both health personnel as well as the patient's own narratives and opinions should be clearly communicated. Knowledge about the patient was presented as more complete in the community setting compared with the knowledge that came from the DPCs. For example, 1 participant concluded:

“In the community, we have followed this patient over the years. We have documents and knowledge about his life and about which treatment works…”

Importantly, our participants reported a discrepancy between the way in which DPCs and community mental health services identified the needs of each patient, separately and from the start, without cooperation.

Staff in inpatient services identify the need for new housing (for the patient) with health personnel present 24 hours a day. With such a high level of care, there is a risk that the patient develops a decreased level of functioning in his/her daily life.

There also seems to be a perceived cultural and power discrepancy between DPCs and the community mental health services. Traditionally, the hospitals have had the “power” to identify the care needed by the patients when discharged. These views seem to have had an influence on the cooperation between systems, with DPCs considered as the most powerful contributors to both treatment and care of the patients.

“We should instead work “shoulder to shoulder”. Now, it is more like the different systems work for themselves.”

Sometimes, patients refuse to engage in the sharing of information. In such cases, community care services struggle to identify the right level of care required.

“In those cases, patients will not establish a relationship with us [community staff] and will not experience our professionalism.”

During the focus group sessions, we found that inpatient staff send information by letter to the community mental health services, a choice of communication method that causes delays in establishing health care in the communities. One participant explained the potential effect of these delays, as follows:

“We could potentially provide health care too late, not knowing that the patient was in need of our services.”

A new e‐message system^33^ seems to have changed the routines for communication between DPCs and community mental health services. As 1 participant puts it:

“It is easier to get documented information when we ask for complementary health information by e‐messages …then, they are obliged to respond.”

Although the e‐message system was introduced to support patient transitions across the healthcare sector, the participants experienced a lack of information and cooperation and stated that, sometimes, they did not get the messages at all.

“What I find scary about e‐messages is that it is like an ordering service, without cooperation. We have to get ready for the service they ordered… but we have waiting lists and a tough prioritization process when deciding who we can help…”

A previous study^34^ identified a lack of communication between DPCs and community mental health services, and the Norwegian Labor and Welfare Administration (NAV) as a significant barrier. The participants in that study pointed out that they could spend hours, days, or even weeks attempting to reach the right person with the authority to make decisions regarding the discharge of patients.

“And we are critical of NAV all the time. We send requests for economic help and support, money for medication, applications for jobs for the patients, or other welfare or coverage of expenses.”

For some patients, attending meetings and gleaning information from these meetings could also be challenging.

“It is as one of the patients always says: There is a big difference depending on the level of sickness. If my anxiety level is high, I remember nothing of what happened there.”

All participants agreed that part of their role is to secure the information given in meetings and inform the patients afterwards, to ensure that they fully understand the decisions made.

Another topic identified in the interviews was the lack of resources needed to give quality mental health care to patients. The participants complained about not having the time and resources at work to prevent the development of mental health problems in their communities.

“Earlier, we had a mental health nurse working on preventing the development of mental illness among children and young people at school. This service is now reduced from three days a week to one day a week.”

In addition, the interviews revealed the negative impact that economic problems in communities had on the training of mental health nurses. One participant expressed her concern with the following remark:

“*The training of the mental health staff is reduced, and that is alarming.”*

The reduced training was deemed to have come about as a cost‐saving initiative, and participants were anxious to hold on to current resources in the face of this and determined to fulfill their duties of care in mental health work, regardless of this context.

### 3.2 Patient activation

Patient activation is considered an important and empowering element in health care reforms. It involves giving patients information that they can understand and act on, and providing them with support that is customized to their needs, so that they are equipped to learn how to manage their own health. Activated patients develop their own understanding of and are engaged in their role in healthcare processes.^35^, ^36^

As evidenced by the interviewees' responses, the community mental health teams emphasized the importance of patient involvement and participation in mental health care. One participant offered the following insight:

“We are making a decision contract together with the patient—what their opinions and goals are—and we have an ongoing dialogue with him/her, to make sure that it is what the patient wants to achieve.”

The very experienced personnel interviewed for this study emphasized that the transition from inpatient status to living in the community could be seen as a challenge for patients.

“The transition to going back into the community with only a few visits every week, is quite overwhelming when you have been together with others 24 hours a day or you could get help 24 hours a day.”

This transition involves patients being discharged from a hospital unit and returning to their homes with less chance to talk to someone around the clock. Unlike the general population, most patients with mental illness live alone, and for some, their social network revolves around those they encounter as part of receiving their health care.^37^

It is not easy for patients to make the transition from living in a safe environment where someone is always available to provide advice, to living at home, where they must try to figure out everything, mostly on their own. Another problem that may arise during the transition phase is that some patients might feel healthy when discharged from hospital‐based services and, therefore, refuse to receive follow‐up care from the community mental health nurses. On some occasions, this could lead to a relapse.

“Some patients think they are healthy and that every problem is solved when they leave the inpatient services; therefore, they don't want follow‐up from any professional personnel… Then, they often have a relapse weeks or months later.”

In the community, the mental health teams work together with the ambulant teams to provide follow‐up care to the patients discharged from the inpatient setting in order to maintain continuity in the provision of mental health care. One participant underscored the importance of providing follow‐up care and of cultivating cooperation between the health care personnel involved:

“When the patients are discharged [from DPC], we think that it is very important [to continue] with visits and treatment from the ambulant team, preferably together with a community mental health nurse.”

Our participants found that coordinated visits to newly discharged patients in the community that involve *both* inpatient and community staff are useful, especially when the patient is new to receiving community mental health services. The staff from the hospital‐based service can introduce the community mental health nurse(s) to the patient, and all 3 parties can discuss the proper treatment and follow‐up.

In addition, the interviews conducted for this study revealed that mental health team members focus not only on the patients but also on their families and settings.

“We support and empower them to improve the patient's function, but in the community, we not only have the patient, we very often also have the whole family, in many different settings.”

During the interviews, the members of the community mental health teams emphasized how challenging it is for patients to cooperate with NAV.

“Many of the patients with whom I have a therapeutic dialogue emphasize that it is a challenge to cooperate with NAV. They don't feel that they are being seen or respected.”

“They are frightened about not fulfilling what is expected from them. Some seem to be afraid that, if they don't say yes to everything, they might lose money or benefits from NAV.”

In addition, NAV's housing policy affects patients' sense of dignity. To have proper housing seems to be an important factor in patients' lives, as evidenced by 1 participant's comment:

“If patients get respectable housing, we see that they begin to flourish and get a new outlook, both on themselves and on their way of life.”

Healthy Life Centres have recently been established as a public health care service in Norwegian communities. They emphasize physical activity and offer counselling, support, and education on issues related to mental health. One participant noted the connection between physical health and mental health:

“Many of the patients struggle with obesity. It is a part of their mental problem. It can also be a side effect of medication. It can be associated with too little activity. We offer a course on diets with a focus on learning how to shop for food and how to make simple, healthy food.”

However, some patients with mental health problems who attend the diet course feel stigmatized because they sense that others attending this open course are watching them with suspicion.

“All kinds of people are participating there, and some of them look down on people suffering with mental problems. Regardless, some patients have attended the course.”

The interviewees also discussed the level of responsibility for training patients with mental health problems in the communities. One participant described how opinions differed regarding this issue:

“We tried to cooperate with the inpatient services to offer a course in coping with depression. We felt that the DPCs were also responsible for training the patients, but the DPCs felt that the communities had to arrange the courses themselves.”

The community mental health nurses seemed to be aware of their role in sharing responsibility for the future training of patients, but they also noted that they lacked the resources to fulfil this role.

“… but we need more professionals, competence, and resources.”

A recent study^38^ showed that the use of peers as co‐educators might contribute to the implementation of a different mental health care delivery system, a system that ensures patient activation and participation in the treatment.

Our participants found it important to have an action plan in place for those patients whose health worsens after discharge from the DPCs. One participant explained the importance of having such a plan, as follows:

“It is necessary to have a plan for readmission to the inpatient services if we observe that patients are not confident and are in need of more security, so they have an opportunity to go back and forth.”

Another participant acknowledged the difficulty encountered by some patients following their discharge:

“Moving back to a house or flat can be quite challenging. Not all patients are capable of coping straight away.”

Our participants were familiar with the allotment of low‐threshold beds (self‐referral admissions) in hospital‐based services/DPCs. This was considered an opportunity for patients to be more involved in their own care.

In relation to clinical care, the participants agreed that teaching patients a range of skills to increase their ability to have a good life in their own home was of utmost importance for success.

We have summed up our findings in Table 1.

**Table 1:** A summary of participant views in the transition process between district psychiatric hospital centres (DPCs) and community mental health services

Main Themes/Categories	Promote Patients Transition	Impede Patient Transition
*Integrated care*		
Information	Opportunity for information sharing	The lack of a single responsible person at each level. Delays in information sharing.
Documentation	Implementation of systematic plans.	The lack of systematic plans.
	Use of e‐messages.	
Team work/ambulant	Around‐the‐clock care.	The lack of meetings.
	One responsible health person in each system for each patient.	Difficulties in identifying the right staff at different care levels.
Resources	Gearing up community services to specialized care.	Lack of specialized personnel.
*Patient activation*		
User involvement and autonomy	Involvement of patients and their families in the admission and treatment process.	
Mutual learning and training	Day centres and healthy life centres that offer counselling, support on issues related to mental health.	Lack of day centres and personnel for training and support.
Relationship		The complexity of welfare systems negatively affected patient dignity.

## 4 DISCUSSION

The main promoting factors affecting smooth care pathways in mental health found in this study were that there should be opportunities for information sharing between inpatient and community mental health services, the identification of health personnel responsible for carrying out the tasks of information sharing and implementation of systematic procedures, the use of digital messages, around‐the‐clock care, and patient involvement. Barriers that prevent the actions described earlier are lack of a responsible person in each level of care; insufficient meetings, protocols and systematic plans; delays in information sharing; and welfare systems negatively impacting on patient dignity.

The mapping of responsible personnel will secure smooth pathways in the transition from being an inpatient to being a user of community mental health care. Our participants also shared their opinions on other important aspects of integrated care.

Patients face challenges in finding their way through the different systems. Patients are in need of support around the clock in order to be activated and empowered to be part of the decision‐making process and develop coping skills.

The gaps between inpatient care and community care appeared when the different services wanted others to be responsible for activities, visits, admission, or new admission to other levels in health care. These gaps were quite evident when participants described differences in opinion between DPCs and community mental health services regarding their respective responsibilities for courses offered to patients with mental health problems. The roles of inpatient and community staff should be clearly delineated so that the different health care services own their respective responsibilities. Participants concluded that improved communication strategies seemed to be the best way of achieving this.

Information seems to be the key to a smooth transition of patients with mental health conditions from inpatient to community facilities. The community mental health team members emphasized the importance of different opportunities to exchange information and their responsibility in providing quality health care, as stated in the Norwegian government's goals for mental health care. If the DPCs confirm that a patient has little need for follow‐up care because of excellent self‐care, there is no need for additional information. However, if the patient has required 24‐hour‐a‐day care and experienced multiple readmissions during the past year, the community personnel need a detailed care plan to avoid serial readmission to hospital‐based care. In particular, our participants pointed out the urgent need for an action plan when patients begin to relapse in the community. Importantly, health personnel involved in deciding the level of care for each patient must take into consideration the comprehensiveness of the written and oral information about their health alongside the social context, resources over time, ongoing psychological symptoms, and the daily functioning of the patient.

The new e‐message system appears to have changed the routine for communication across DPCs and community services, providing more complementary health information. However, these are also subject to a lack of cooperation and failure to receive messages. That said, experiences from a recent study in Norway^33^ showed that electronic messaging is more efficient and less time‐consuming than previous means of communication and is considered to be a useful tool for communication and collaboration in patient transitions.

Patients sometimes refused to share information about their health and, consequently, community services had difficulties in choosing the right level of care. With systematic written procedures and documentation, it would be much easier for community personnel to find out what has or has not been done, and the randomness in the process of being transferred as a patient from 1 system to another, would decrease. This is in line with Durbin et al,^31^ who suggested that the use of structured forms to share information could have a positive effect on the necessary flow of information and possibly reduce the time spent on finding the right people in the various systems.

The pathways of care seem to be a bureaucratic process, resulting in difficulties for patients wanting to complain if they find their legal rights to be compromised. Although the decisions are made on the basis of the knowledge of each discipline and on the economic resources available to provide equal treatment for patients, the knowledge of the different disciplines should be accorded greater weight than the economic resources available in decisions related to care.

The shift in specialized care from hospitals to communities is part of a trend to promote discharge from hospitals at the earliest possible stage. For this to succeed, there is a need for sufficient staffing levels of specialized health personnel—in inpatient services—focused more on treatment, and community contexts, focused more on care. A study in Norway^39^ on care pathways in mental health care highlighted the important contextual knowledge of each kind of health service. However, care pathways could become regulation tools that limit professional autonomy and devalue contextualized knowledge.

The participants also described increased patient satisfaction and motivation to receive care when they are more fully involved in the admission and treatment process. This finding is in line with Tveiten et al,^40^ who advised giving patients a voice to express their concerns and have these addressed. In addition, a recent study in the UK^1^ showed a loss of the patient's voice at the key transition points into and out of acute inpatient mental health care. Moreover, as reported earlier,^34^ the establishment of relationships among the 3 parties involved (patients, inpatient staff, community staff) was considered to be of utmost importance in the transition process between inpatient and community mental health care.

Participants reported that health personnel tried to involve patients to a greater degree in the decisions concerning their health care and future plans. However, a shared decision‐making process can be a difficult experience for some patients, especially those who have cognitive difficulties because of their illness. Health care professionals need to identify to what degree patients want to be part of the decision‐making process, but, as a main rule, a shared approach to this should be promoted as first choice, when appropriate.^41^, ^42^

Research has provided evidence of the benefits of greater patient involvement.^43^ A recent study^44^ about patients' knowledge and the power imbalance in the doctor‐patient relationship supports our findings that patients need knowledge and power to participate in a shared decision‐making process. However, a discourse analysis of the concept of patient involvement in mental health nursing in the UK^45^ pointed out the implications for the role of mental health nurses and concluded that nurses may need to relinquish power to patients if true involvement is to occur.

Some of the communication strategies to meet the needs of patients should focus on a better sharing of knowledge through enhanced teamwork and interprofessional collaboration. Annells et al^46^ found that the sharing of knowledge ensured an effective referral process. This finding was also described by Beach and Oates,^47^ who found that a key aspect of the work of mental health nurses is sharing information about individuals through records. They concluded that shared information through electronic records reduces unnecessary documentation and increases collaboration and the quality of direct care. Our participants described general practitioners as the most important collaborating partners for community mental health nurses. In addition, our participants called for improved therapeutic communication skills among providers of somatic home care, as well as closer cooperation with somatic home‐care services.

The participants also emphasized that it is no longer easy for chronically ill patients to be granted admission to inpatient facilities due to the policy that most of the treatment should be in the patients home instead of in hospital. So there seems to be a discrepancy between the policy and the needs in the communities. It would be interesting to explore the patients views on this matter. Communities with economic problems are struggling to provide the resources and further training necessary to ensure that patients receive quality mental health care. Finally, there should be less emphasis on developing and enforcing bureaucratic rules and regulations for health care, and more emphasis on producing competent professional health personnel and on providing help to patients around the clock. This shift in emphasis is an approach that could be less costly when measured over time. More research should also be conducted on the effectiveness and efficiency of the planning of care pathways from a longer‐term perspective than that of the current hospital/community admission process. Patients will probably be more compliant with treatment if they participate in the decision‐making process, in accordance with their rights.

### 4.1 Limitations and strengths of the study

The findings of our qualitative study are non‐generalizable but offer valuable insights and understanding about the phenomena of care pathways in the transition between inpatient DPCs and community mental health services. We would like to point out that our national health system could be different from other countries. Despite the small sample size, we derived a rich and contextualized information from key personnel about promoting factors and barriers in the care pathways for this transition. Such findings can assist in tailoring the organization of care pathways to enhance the patient experience of mental health care transfers. We acknowledge that our focus has been the health planning system in a region in Norway and different findings may emerge from other regions in this country and other countries. Our findings indicate that further and more comparative research could test and build upon these initial findings.

## 5 CONCLUSION AND RECOMMENDATIONS

The mapping of responsible personnel will secure the follow‐up of the key findings in the point of transition between services, in terms of cooperation, information, and documentation.

To ease the transition for patients leaving around‐the‐clock treatment and care and reentering the community, it is important to secure proper follow‐up at the right time. If communication fails, people in need of re‐admission might not be identified.

A setting with a single responsible person (and system) and clarified procedures should be implemented at each stage in care pathways to avoid waivers of liability and to provide transparent systems that can be easily monitored by health personnel and patients. Such a person could be responsible for coordinating services as well as liaise between social‐ and health systems and patients.

Both digital and telephonic sharing of information and communication should be implemented and in place before admission to a hospital‐based service, and before and after discharge back to the community. In order to secure effective information sharing, all parties should have the phone number of a named, responsible coordinator in each health care and social care system to allow easy access to all parties. Regular meetings should be scheduled, in which mental health personnel can share and discuss key information with the social care system, to avoid the long current delays that extend inpatient status and block satisfactory transition to the community setting.

## CONFLICT OF INTEREST

None declared.

## AUTHOR CONTRIBUTIONS

Conceptualization: Valentina C. Iversen, Eva W. Sather, Marit F. Svindseth
Formal analysis: Valentina C. Iversen, Eva W. Sather, Marit F. Svindseth
Writing—review and editing: Eva W. Sather, Marit F. Svindseth, Valentina C. Iversen, Paul Crawford
Writing—original draft: Eva W. Sather
